# Ultrathin and Ultrastrong Kevlar Aramid Nanofiber Membranes for Highly Stable Osmotic Energy Conversion

**DOI:** 10.1002/advs.202202869

**Published:** 2022-07-03

**Authors:** Li Ding, Dan Xiao, Zihao Zhao, Yanying Wei, Jian Xue, Haihui Wang

**Affiliations:** ^1^ School of Chemistry and Chemical Engineering South China University of Technology Guangzhou 510640 P. R. China; ^2^ Beijing Key Laboratory for Membrane Materials and Engineering Department of Chemical Engineering Tsinghua University Beijing 100084 P. R. China

**Keywords:** ion selective, Kevlar aramid nanofiber, membrane, nanofluidic, osmotic energy

## Abstract

An ion‐selective membrane can directly convert the osmotic energy to electricity through reverse electrodialysis. However, developing an advanced membrane that simultaneously possesses high power density, excellent mechanical strength, and convenient large‐scale production for practical osmotic energy conversion, remains challenging. Here, the fabrication of ultrathin and ultrastrong Kevlar aramid nanofiber (KANF) membranes with interconnected three‐dimensional (3D) nanofluidic channels via a simple blade coating method is reported. The negatively charged 3D nanochannels show typical surface‐charge‐governed nanofluidic ion transport and exhibit excellent cation selectivity. When applied to osmotic energy conversion, the power density of the KANF membrane‐based generator reaches 4.8 W m^–2^ (seawater/river water) and can be further increased to 13.8 W m^–2^ at 328 K, which are higher than most of the state‐of‐the‐art membranes. Importantly, a 4‐µm‐thickness KANF membrane shows ultrahigh tensile strength (565 MPa) and Young's modulus (25 GPa). This generator also exhibits ultralong stability over 120 days, showing great potential in practical energy conversions.

## Graphical abstract

An ultrathin and ultrastrong Kevlar nanofiber (KANF) membrane with interconnected three‐dimensional nanofluidic channels is prepared for long‐term stable and high‐performance osmotic energy conversion, the power density of the KANF‐based generator reaches 4.8 W m^–2^ (seawater/river water) and  up to 13.8 W m^–2^ at 328 K. This environmentally friendly, and robust membrane holds great potential for practical energy conversions.

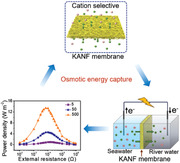

## 1 Introduction

Mixing salt water and fresh water generates heat according to the Gibbs' free energy. However, this energy can be directly converted into electricity not only for domestic and industrial use (e.g., osmotic energy conversion between sea water and river water),^[^ ^1^ ^]^ but also provide power for implant devices such as heart pacemakers, prosthetic units and small portable power supplies, which is considered as a renewable, clean, and sustainable energy source.^[^ ^2^, ^3^, ^4^ ^]^ Gibbs’ free energy capture requires an ion‐selective membrane to separate the salt water and the fresh water in a reverse electrodialysis (RED) process.^[^ ^5^, ^6^, ^7^ ^]^ One of the most pressing issues in osmotic energy harvesting is the development of high‐performance and efficient ion‐selective membranes.^[^ ^8^, ^9^, ^10^, ^11^, ^12^, ^13^, ^14^ ^]^ Generally, an ideal membrane should possess high power density, easy scale‐up, and sufficient mechanical strength for practical operation.^[^ ^15^, ^16^ ^]^ Traditional membranes in RED systems are the ion exchange membranes used in electrodialysis processes. However, they are limited by the poor output power density and their high resistance.^[^ ^5^ ^]^ Recently, inspired from biological ion channels and with the development of synthesis techniques, membrane with nanofluidic ion channels have been extensively investigated explored.^[^ ^17^, ^18^, ^19^ ^]^ In nanofluidic channels, the ion transports exhibit significantly different properties from that of the bulk solutions, in which the surface charge of the channels governed the ionic behaviors.^[^ ^20^, ^21^, ^22^ ^]^ The charged wall attracts ions with the opposite charge (counter ions) and excludes ions with the same charge (co‐ions), thus the counter ions preferentially transport over the co‐ions, generating the ion selectivity. In nanofluidic channel with dimensions closing to the Debye screening length, the electrical double layers (EDLs) are usually overlapped, representing unipolar ionic transport. Such unipolar ionic transport can enhance the ion selectivity and contribute to a higher net diffusion current.^[^ ^23^, ^24^ ^]^ For example, Radenovic et al. demonstrated that the nanoporous single‐layer molybdenum disulfide (MoS_2_) based generator can produce an ultrahigh power density of up to 10^6^ W m^−2^.^[^ ^25^ ^]^ However, these nanopore‐based membranes generally suffer from the drawbacks of elusive scalability and limited pore controllability.^[^ ^15^ ^]^ The newly fashioned bioinspired asymmetric membranes, composed of ion selective and supporting layers, have realized high output power densities due to their advanced structural design.^[^ ^26^, ^27^, ^28^, ^29^, ^30^ ^]^ However, the selective layers of these composite membranes are still confronted with mechanical problems and low porosity, restricting their practical applications.^[^ ^15^ ^]^ The development of large‐scale robust ion selective membranes with the simultaneously high power density and convenient mechanical properties is still challenging.

In this work, we have processed Kevlar (poly(p‐phenylene terephthalamide)) nanofibers (KANF), a star material that possesses the merits of good mechanical properties and low cost.^[^ ^31^ ^]^ Kevlar yarns are famous for their applications in body armor and ultrastrong p‐aramid synthetic macroscale fibers with a high tensile strength‐to‐weight ratio.^[^ ^32^, ^33^ ^]^ Recent studies have demonstrated that by weakening the interactions between neighboring aramid chains through hydrogen bonds, the Kevlar yarns can be split into nanofibers with a diameter of 5–30 nm and a length of up to tens of microns.^[^ ^34^, ^35^ ^]^ The obtained nanofiber solution can be further used to fabricate membranes with three‐dimensional (3D) interconnected nanochannel networks, which have been emerged as new nanoscale building blocks for selective membranes in nanofiltration and desalination.^[^ ^34^, ^35^ ^]^ During the splitting process, abundant new oxygen‐containing functional groups (such as carboxyls) are generated on the nanofiber, providing a negatively charged surface.^[^ ^36^, ^37^ ^]^ Therefore, KANF membranes hold the potential for osmotic energy conversion. Recently, although several reports that used KANF as additives in the composite membrane to improve the osmotic energy conversion efficiency, the obtained power density is still undesirable.^[^ ^9^, ^10^, ^36^, ^38^ ^]^ To the best of our knowledge, researches about ultrathin and ultrastrong pure KANF membranes for osmotic energy conversion are rarely reported.

**Figure 1:**
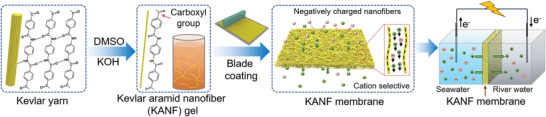
Scheme of the preparation of KANF membrane‐based nanofluidic osmotic energy harvesting device. The Kevlar yarns can be split into nanofibers by using the mixed solution of DMSO and KOH. Through a blade coating method, a free‐standing and robust KANF membrane can be obtained. Because of the existence of carboxyl groups on KANFs, the prepared KANF membrane is cation‐selective. The KANF membrane was constructed in the two‐compartment electrochemical cell to harvest osmotic energy from ionic solutions with different salinity gradient.

Herein, we report an industrially adaptable method to prepare a large‐scale, ultrathin, and ultrastrong KANF membrane‐based generator for high‐efficient osmotic energy conversion (**Figure** **1**). The fabrication process is based on a simple blade coating method. Benefiting from the interconnected 3D nanofluidic channels and the hydrogen bonds between the nanofibers, the KANF membrane not only shows high ionic conductance and excellent cation selectivity, but also exhibits outstanding mechanical strength. The blade coating method is scale‐up friendly and can easily produce membrane areas of the order of several hundred square centimeters. By mixing synthetic seawater and river water, the output power density of a 2‐µm‐thick KANF membrane‐based energy generator reaches ≈4.8 W m^–2^. Besides, the power density can be further increased up to 13.8 W m^–2^ at 328 K. Importantly, the KANF exhibits ultralong working stability over 120 days, which is attributed to the intrinsic chemical resistance of the nanofibers. The achieved top‐level tensile strength of the KANF membrane is 565 MPa, which is the highest among the reported membranes with similar thickness.^[^ ^19^, ^36^, ^39^, ^40^, ^41^, ^42^, ^43^, ^44^, ^45^, ^46^ ^]^ Our work not only shows the great potential of the KANF membrane for large‐scale salinity gradient energy conversion, but also paves the way for the applications of the KANF membrane in ion sieving, nanofluidics and membrane separations.

## 2 Results and Discussion

**Figure 2:**
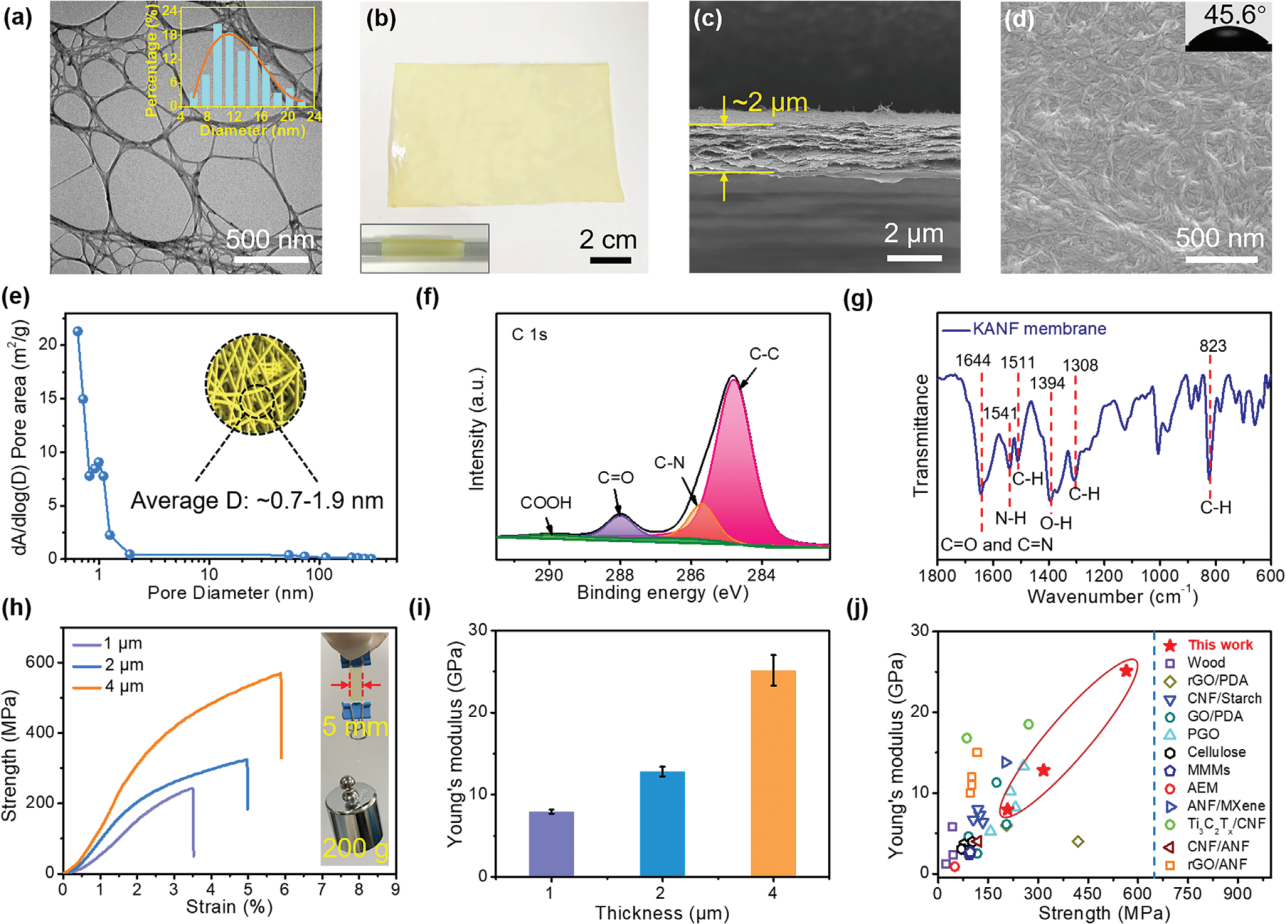
Characterizations of KANF membrane. a) TEM image of the KANFs and the diameter distribution (figure inset). b) Free‐standing and transparent KANF membrane and its crimping state (figure inset). c,d) Cross‐sectional and surface SEM images of the KANF membrane, respectively. e) Pore size distribution of the KANF membrane derived from BET result. f) XPS result of the high‐resolution spectrum of C 1s of the KANF membrane. g) FTIR spectrum of the KANF membrane. h,i) Tensile strength curves and the Young's modulus of the KANF membrane with different thicknesses, respectively. The inset of (h) shows that a 2‐µm‐thick and 5‐mm‐wide membrane can carry a weight of 200 g. Error bars represent s.d. j) Comparison of the mechanical properties of the KANF membrane with the state‐of‐the‐art membranes.

We obtain a dark red, viscous solution by dissolving the crude fiber (Figure S1, Supporting Information) in a DMSO/KOH solution for one week at room temperature. The Kevlar yarns can be split into nanofibers (Figure S2, Supporting Information) by weakening the hydrogen bonds and simultaneously strengthening the electrostatic repulsion between the polymer chains.^[^ ^35^ ^]^ TEM image shows that the diameters of the nanofibers are about 8–20 nm, and their lengths are tens of microns (**Figure** **2**a). The corresponding elemental mapping indicates the presence of the elements C, N, and O (Figure S3, Supporting Information). The nanofiber solution was then cast onto a glass plate by using a doctor blade (Figure S4, Supporting Information), which can be extended to a large scale and continuous production. After thermal treatment, a free‐standing, transparent, and pale‐yellow nanofiber membrane can be easily obtained (Figure 2b). The cross‐sectional SEM image indicates that the thickness is ≈2 µm (Figure 2c). The surface SEM image of the nanofiber membrane shows interconnected nanofibers without any defects (Figure 2d). The water contact angle indicates that the nanofiber membrane has a hydrophilic surface (Figure 2d inset). The surface roughness of the nanofiber membrane was characterized by atomic force microscopy (AFM) (Figure S5, Supporting Information). Average surface roughness of ≈6 nm demonstrates a smooth surface of our nanofiber membrane. The BET result shows that the pore size distribution of the KANF membrane is around 0.7–1.9 nm (Figure 2e). The membrane's thickness can be tuned by adjusting the gap between the blade and the substrate (Figure S6, Supporting Information).

The chemical composition of the nanofiber membrane is further investigated by X‐ray photoelectron spectroscopy (XPS) (Figure 2f and Figure S7, Supporting Information). The high resolution C 1s spectrum could be fitted into four peaks, which are assigned to C–C (284.8 eV), C–N (285.7 eV), C=O (288.0 eV), and −COOH (290.0 eV). Further, the N 1s spectrum confirmed the existence of amide bonds (N—C) at 400.1 eV, which is consistent with previous work.^[^ ^47^ ^]^ The Fourier‐transform infrared spectroscopy (FTIR) further reveals the chemical structure of the KANF membrane. The result (Figure 2g) shows the stretching frequency of the carboxyl groups (1644 cm^–1^) in amides, the bending frequencies of the amides (1541 cm^–1^), the O−H bending vibration (1394 cm^–1^) and the aromatic C—H bonds in the benzene rings. The mechanical property of a membrane is crucial for its practical applications.^[^ ^15^ ^]^ The free‐standing KANF membranes also exhibit excellent mechanical strength for practice handling (Figure 2h,i). The tensile strength of a ≈1‐µm‐thick nanofiber membrane is ≈208 MPa, with a Young's modulus of ≈8 GPa. By increasing the thickness of the membrane to ≈4 µm, tensile strength is improved to ≈565 MPa, while the Young's modulus reaches ≈25 GPa. To the best of our knowledge, these values are the highest among the reported membranes with similar thicknesses (Figure 2j). For a visual demonstration (Figure 2h inset), a 2‐µm‐thick, 5‐mm‐width KANF membrane can withstand a 200 g weight easily. These results suggest that our KANF membrane with impressive mechanical strength, is suitable for practical osmotic energy conversions. We attribute the excellent mechanical strength of KANF membrane to the regeneration of hydrogen bonds of the crosslinked nanofibers during thermal treatment and the superiority of blade‐coating method. which have demonstrated by previous studies.^[^ ^45^, ^48^, ^49^ ^]^ To further prove the superiority of blade‐coating method, we also prepared the KANF membrane through vacuum filtration method. As shown in Figure S8 (Supporting Information), we can see the tensile strength of filtrated membrane is ≈104 MPa, five times lower than that of the blade‐coated membrane.

**Figure 3:**
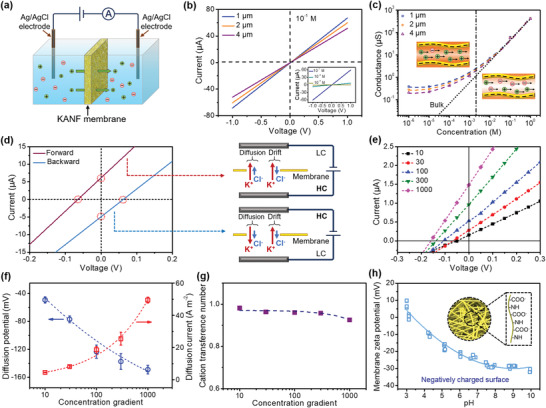
Ionic transport properties of the KANF membrane. a) Schematic of the ion transport measurement. b) *I–V* curves of the KANF membrane with different thicknesses and different KCl concentrations (inset). c) Ionic conductance of the KANF membrane as a function of electrolyte (KCl) concentration. When the concentration is <10^–3^ m, the tested conductance gradually deviates from the bulk solutions, indicating the surface‐charge‐controlled ionic transport behavior. The insert pictures illustrate the ion transport in simplified nanochannels under different concentrations. d) The forward and backward diffusion *I–V* curves of the KANF membrane were measured under the concentration gradient of a 50‐fold KCl and the corresponding schematic illustration of the experimental setup. The direction of the diffusion current is consistent with the net flow of cations from high to low concentration, demonstrating that the KANF membrane is cation selective. e) The *I–V* curves of the KANF membrane under a variety of KCl concentration gradients. f) The generated diffusion potential and diffusion current as a function of the KCl concentration gradient. Error bars represent s.d. g) Cation transference number (*t*_+_) of the KANF membrane versus concentration gradient. h) Membrane zeta potential of the KANF membrane as a function of pH. The inset shows the negatively charged groups on the nanofiber.

The KANF membrane was further clamped in a two‐chamber electrolytic cell to investigate the ions’ transportation properties (**Figure** **3**a). A pair of Ag/AgCl electrodes was used to test the current–voltage (*I–V*) curves across the membrane in KCl electrolyte with various concentrations. Figure 3b shows the corresponding *I–V* curves of the nanofiber membranes with thicknesses of 1, 2, and 4 µm, respectively. With the increase of membrane thickness, the ionic current decreased from 64 to 50 µA at the voltage of 1 V, which is attributed to the increasing resistance. Furthermore, we measured the ionic conductance of the KANF membrane as a function of KCl concentration (Figure 3c). The transmembrane conductance gradually deviated from the bulk value (dashed line) when the KCl concentration is lower than 0.01 m, and turns to a plateau at low concentrations (<10^–4^ m), which is due to the surface‐charge‐governed ion transportation.^[^ ^9^, ^50^, ^51^ ^]^ The insert pictures (Figure 3c, inset) illustrate the ion transport in simplified nanochannels under different concentrations. In the high‐concentration region, the length of the EDLs (*λ*_D_) is lower than the average channel size,^[^ ^52^ ^]^ resulting in a bulk‐like ion diffusion. While in the low‐concentration region, *λ*_D_ is close to the diameter of the channel, which results in an overlapping EDLs, thus the ion transport is controlled by the surface charge.

We also demonstrated that the KANF membrane is cation selective. The KANF membrane was immersed in KCl solution (0.1 m) for 1 h and then washed with deionized water to remove the residual ions. The corresponding SEM energy‐dispersive spectroscopy (EDS) mapping results show that the intensity of the element K is significantly larger than that of the element Cl (Figure S9, Supporting Information), demonstrating that the KANF membrane has a preferential cations attraction.^[^ ^53^ ^]^ Further, to investigate the ion selectivity, the *I–V* curves were conducted with various KCl concentration gradients over the membrane. KCl was selected as the representative electrolyte because of the very similar bulk mobilities of K^+^ and Cl^–^ ions. The forward and backward diffusion *I–V* curves of the KANF membrane measured under a 50‐fold KCl concentration gradient and the corresponding schematic illustration of the experimental setup are shown in Figure 3d. The ions diffuse across the membrane spontaneously under the concentration gradient. It has been demonstrated that the diffusion current is generated by the separation of cations and anions in the electric double layer at the inner wall of the nanochannel, which can be explained by the coupled Poisson and Nernst–Planck equations.^[^ ^54^ ^]^ The open‐circuit potential *E*_oc_ (the potential at zero current) and the short‐circuit current *I*_sc_ (the current at zero external voltage) could be derived from the intercepts of the experimental *I–V* curves (Figure S10, Supporting Information). It should be noted that *E*_oc_ consists of two parts (Figure S11, Supporting Information): the redox potential (*E*_redox_) generated by the unequal potential drop at the electrode–solution interface in different electrolyte concentrations, and the diffusion potential (*E*_diff_) contributed by the membrane.^[^ ^36^, ^52^ ^]^ The direction of the diffusion current is consistent with the net flow of cations from high to low concentration (Figure 3d inset), further demonstrating that the KANF membrane is cation selective.^[^ ^55^ ^]^ We also measured the *I–V* curves of the KANF membrane under a variety of KCl concentration gradients (Figure 3e), with the high‐concentration (HC) side was ranging from 1 mm to 1 m, while the low‐concentration (LC) side was fixed at 0.1 mm. As shown in Figure 3f and Table S1 (Supporting Information), the *E*_diff_ increases from ≈57 to ≈151 mV with increasing concentration gradients. When *C*_high_ */C*_low_ = 1000, the *I*_sc_ can reach 49.5 A m^–2^.

The ion selectivity of the membrane as the difference in the diffusive fluxes of cations and anions can be calculated from *E*_diff_ by using equation S1 (Supporting Information).^[^ ^10^, ^27^, ^56^ ^]^ From the data listed in Table S2 (Supporting Information), the transference numbers *t*_+_ under various concentration gradients can be obtained, which are directly related to the fluxes of cations, and can be used to quantify the ion selectivity for cations. As shown in Figure 3g, all calculated *t_+_* exceed 0.90 for all salinity gradients. When *C*_high_/*C*_low_ = 1000, *t_+_* still reaches 0.93, demonstrating the excellent ion selectivity of our nanofiber membrane, which is comparable to the state‐of‐the‐art membrane materials reported in the literatures.^[^ ^36^, ^50^, ^56^, ^57^ ^]^ Additionally, the cation selectivity of the KANF membrane was further verified by measuring the surface streaming potential. The result (Figure 3h) shows that the zeta potential of the KANF membrane is −25 mV (pH ≈ 7), indicating the negatively charged surface of the membrane.^[^ ^58^ ^]^ This negative surface is due to the de‐protonation of the oxygen‐containing surface groups (Figure 3h, inset), which has been verified by the FTIR result. Therefore, the negative surface charges on the nanochannels would attract the counter ions and exclude ions of the same charge, making the KANF membrane a cation‐selective barrier.

**Figure 4:**
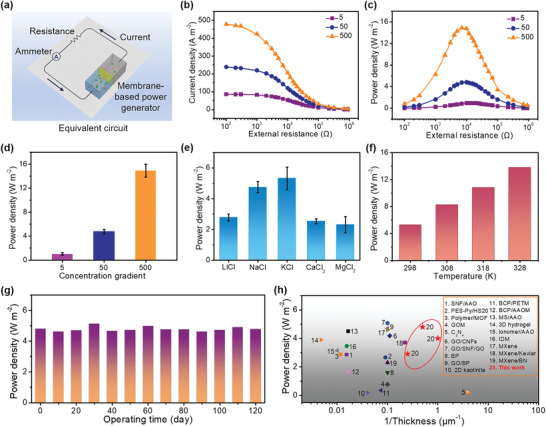
Osmotic energy conversion behavior of the KANF membrane‐based power generator. a) The equivalent circuit of the generator to supply an external resistance. b) Current density and c) corresponding power density of the KANF membrane as functions of the load resistance under three salt concentration gradients. d) The output power density increases with increasing concentration gradients and reaches a maximum value of ≈15.0 W m^–2^ at the 500‐fold concentration gradient. e) Output power density of the membrane under different electrolytes. Error bars represent s.d. f) Output power density of the KANF membrane under different temperatures. g) The KANF membrane‐based power generator shows ultralong operating stability over 120 days. h) Energy conversion performances of the KANF membranes compared with state‐of‐the‐art membranes.

To evaluate the osmotic energy conversion performance of the KANF membrane, the membrane‐based generator was used to supply an electric load resistance (*R*_L_) in an external circuit (**Figure** **4**a). The output power can be calculated as *P* *= I* ^2^ *×* *R*_L_, where *I* is the generated current. The high‐salinity solution was varied from 0.05 to 5 m NaCl, while the low‐salinity solution was fixed as 0.01 m NaCl (Figure 4b,c). Accordingly, the measured current decreases with the increasing load resistance, while the obtained diffusion current and output power density increase as the concentration gradient increases from 5‐ to 500‐fold. Specifically, by mixing synthetic seawater (0.5 m NaCl) and river water (0.01 m NaCl), we can obtain the corresponding *E*_diff_ and *I*_diff_ being ≈67 mV and ≈5.4 µA (the redox potential of the electrode has been subtracted), while the output power density reaches a maximum value of 4.8 W m^–2^ at the load resistance of 10 kΩ. This means that the internal resistance of the membrane is only ≈10 kΩ, which is much lower than those of the membranes reported in literatures.^[^ ^12^, ^15^, ^56^ ^]^ The power density can reach ≈15.0 W m^–2^ under a 500‐fold salinity gradient (Figure 4d) (mimicking salt lake to river water).^[^ ^27^ ^]^ Theoretically, a thinner membrane would produce a higher power density because of the faster ion diffusion, but actually, the inevitable defects of the thin membranes also influence the output power (Figure S12, Supporting Information). Besides, we also found that the finer nanofibers benefit for a higher power density (Figures S13 and S14, Supporting Information).

We also investigated the energy conversion behavior of the KANF membrane with different electrolytes. As shown in Figure 4e, for the monovalent ions, the output power densities of LiCl, NaCl, and KCl, are 2.8, 4.8, and 5.3 W m^–2^, respectively. The highest power density for KCl is attributed to the larger diffusion coefficient of K^+^ in comparison with Li^+^ and Na^+^ inside the KANF membrane (Figure S15, Supporting Information). It has been demonstrated that a faster cation diffusion would lead to a more efficient charge separation for a cation‐selective membrane.^[^ ^59^, ^60^ ^]^ For the divalent ions, such as Ca^2+^ and Mg^2+^, the obtained output power densities (2.5 and 2.3 W m^–2^) are lower than those of the monovalent ions, which can be ascribed to their much lower diffusion coefficients.^[^ ^10^ ^]^ Furthermore, we investigated the effect of temperature on ionic transport and the corresponding impact on output power generation of the KANF membrane. As shown in Figure S16 (Supporting Information), the *I*_sc_ increases with rising temperature from 298 to 328 K. The increased *I*_sc_ is attributed to the enhancement of ionic mobility at higher temperatures.^[^ ^61^ ^]^ The corresponding power density can reach 13.8 W m^–2^ at 328 K (Figure 4f and Figure S17, Supporting Information). The thermal performance widens the practical application of the KANF membrane and offers an approach to boost osmotic power conversion. Besides pure electrolyte solution, the KANF membrane also shows a high power density up to 5.2 W m^–2^ under the real water sources (Figure S18, Supporting Information), where the seawater is obtained from *South China Sea* (≈0.58 m NaCl) and river water is taken from *Pearl River* (≈0.006 m NaCl). The obtained power density of 5.2 W m^–2^ is higher than the benchmark for commercialization (≈5 W m^–2^).^[^ ^62^ ^]^ Excitingly, we found that the KANF membrane exhibits a splendid stability. The osmotic power performance of the KANF membrane can be maintained stable even after 120 days (Figure 4g), which is ascribed to the intrinsic chemical stability of the nanofiber. Importantly, the KANF membrane is still robust enough and maintained chemical stability even after temperature rise and long‐term stability test. By comparison (Figure 4h and Table S3, Supporting Information), we can find that the power density of the KANF membrane‐based generator is higher than most of the state‐of‐the‐art membranes with similar thickness.^[^ ^9^, ^10^, ^12^, ^19^, ^27^, ^30^, ^36^, ^50^, ^51^, ^52^, ^53^, ^57^, ^63^, ^64^, ^65^, ^66^, ^67^, ^68^, ^69^, ^70^, ^71^, ^72^, ^73^ ^]^

**Figure 5:**
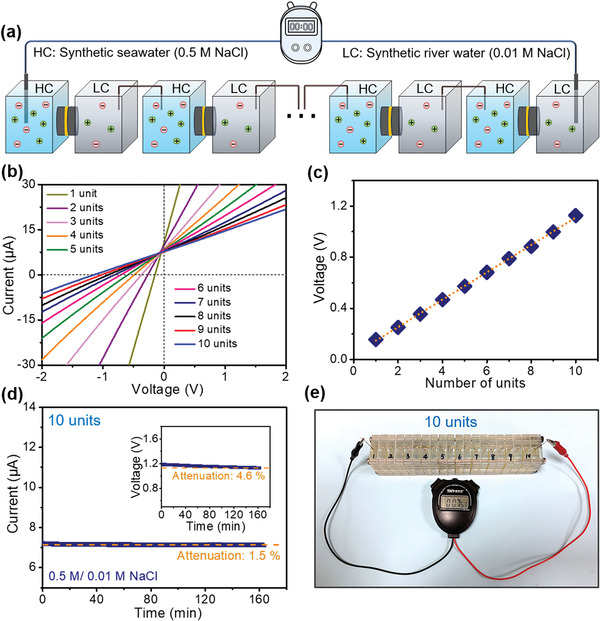
Power output of the tandem stacks of the KANF membrane‐based power generator. a) Scheme of the tandem stacks of the generator. b) *I*–*V* curves of tandem stacks of the KANF membrane‐based power generators (from 1 unit to 10 units). c) The generated voltage of the power generator as a function of the number of units. d) The produced current and voltage of the 10‐units generator with continuous working over 160 min. e) Ten units of the KANF membrane‐based power generators can power a chronometer.

Aiming to further boost the application of osmotic energy conversion, we also constructed a tandem KANF membrane‐based generator to explore the performance of the membrane (**Figure** **5**a). Figure 5b shows the *I–V* curves of 1–10 units of the generator under the salinity gradient of synthetic seawater (0.5 m NaCl) and river water (0.01 m NaCl). The corresponding output voltages exhibit a perfect linear relationship as a function of the stacked units, each single unit cell can produce a voltage of 120 mV (Figure 5c). Without replenishing the electrolyte, the obtained current of the tandem 10 units only exhibits a 1.5% attenuation over 160 min and also a low attenuation of the output voltage (Figure 5d), demonstrating that the KANF membrane‐based generator can continuously provide electroenergy from osmotic energy.^[^ ^36^ ^]^ In addition, the tandem stacks of the generator can directly power a chronometer (Figure 5e).

It should be noted that the testing area of the membrane is about 0.03 mm^2^, which is the same as previous reports.^[^ ^12^, ^15^, ^27^, ^30^, ^36^, ^51^, ^66^ ^]^ A large number of studies show that the power density of nanofluidic membrane for osmosis energy decays with the increase of testing membrane size, which can be ascribed to the combined effect of multiple factors in multi‐scale (for example, the increased reservoir resistance and reservoir/nanopores interfacial resistance, and the increased stochastic physical defects within the membrane).^[^ ^36^, ^74^ ^]^ To this end, we also evaluated the power density of the KANF membrane with the increasing of the testing area (Figure S19, Supporting Information). When the area was increased to 3.14 mm^2^, the power density was decreased to 0.8 W m^–2^, but still higher than that of the state of the art membranes used the same testing area.^[^ ^9^, ^12^ ^]^ Nonetheless, researchers have demonstrated that several technical improvements, such as optimizing the membrane thickness, maximizing the surface charge, and eliminating concentration polarization, can be used to eliminating the entering resistance of the large‐size membrane‐based osmotic power generator and maintaining the high power density.^[^ ^36^, ^74^ ^]^ As for the practical application, the cost of the membrane is an important factor. It can be noted that the raw material cost for 1 m^2^ KANF membrane is around 1.5 RMB (Table S4, Supporting Information), showing great potential for large scale production.

## 3 Conclusions

Robust KANF membranes have been prepared for high‐performance osmotic energy harvesting through a scale‐up friendly blade coating method. Benefiting from the interconnected nanofiber networks and the hydrogen bond between the nanofibers, the KANF membrane shows excellent mechanical properties with a tensile strength up of to 565 MPa. The negatively charged surface, interconnected 3D nanofluidic channels, and ultralow internal resistance ensure the membrane with considerable nanofluidic ion‐transport properties. The KANF membrane‐based generator exhibits an excellent power density of 4.8 W m^–2^ by mixing synthetic seawater and river water. Through raising the working temperature, the output power density can further increase to 13.8 W m^–2^ at 328 K. Owing to the excellent chemical stability of the nanofibers, the KANF membrane shows extraordinary working stability over 120 days. Ten tandem stacks of the generator can directly power electronic devices. The controlled and straightforward process of membrane fabrication, low cost and long‐term stability provide the potential for industrial production. Our work opens up a promising avenue toward large‐scale osmotic energy conversion and potentially spark further applications of such membranes in ion sieving, desalination, and nanofluidics.

## Conflict of Interest

The authors declare no conflict of interest.

## Supporting information

Supporting Information

## Data Availability

The data that support the findings of this study are available from the corresponding author upon reasonable request.
