# Supplementary material for: Ultrathin and Ultrastrong Kevlar Aramid Nanofiber Membranes for Highly Stable Osmotic Energy Conversion
Source: Adv Sci (Weinh). 2022 Jul 3;9(25):2202869. doi: 10.1002/advs.202202869 (PMC9443462; doi:10.1002/advs.202202869)
Supplement: Supplementary file 1 — Supporting Information [file ADVS-9-2202869-s001.pdf]

ADVANCED SCIENCE
Open Access

Supporting Information

for *Adv. Sci.*, DOI 10.1002/advs.202202869

Ultrathin and Ultrastrong Kevlar Aramid Nanofiber Membranes for Highly Stable Osmotic Energy Conversion

Li Ding, Dan Xiao, Zihao Zhao, Yanying Wei, Jian Xue and Haihui Wang**

Supporting Information

Ultrathin and ultrastrong Kevlar aramid nanofiber membranes for highly stable osmotic energy conversion

Li Ding, Dan Xiao, Zihao Zhao, Yanying Wei, Jian Xue, and Haihui Wang**

***Corresponding authors. E-mail: xuejian@scut.edu.cn; cehhwang@tsinghua.edu.cn.

Methods

Synthesis of KANF nanofibers and KANF membranes

The KANF membrane was synthesized as follows.[1] 2 g of Kevlar yarns (purchased from Dupont) and 2 g KOH (purchased from Aladdin) were added into 100 mL of DMSO (purchased from Aladdin). The mixture was magnetically stirred for 1 week at room temperature. By this method, the strong bond interaction between crude fibers molecular chains, namely hydrogen bond, π-π bond and van der Waals force can be decomposed, and large-scale fibers can be transformed into nanofibers. After stirring for 7 days, the solution gradually turns dark red and becomes a viscous solution. The obtained KANF dispersion was then poured on 0.1 μm organic nylon substrate to prepare the KANF membrane by the blade coating method, and the KANF membrane of different thicknesses can be obtained by changing the height of the scraper. After vacuum drying, the KANF membrane can be easily removed from the substrate to form an ultra-thin and free-standing KANF membrane.

Characterization of the KANF dispersions and membranes

SEM images were obtained using a Hitachi SU8220 device. The SEM elemental mapping analysis was conducted using an energy-dispersive X-ray spectrometer (Oxford EDS, with INCA software). TEM images were obtained from JEOL JEM-2100F device with an acceleration voltage of 20 kV. TEM mapping was conducted using the Bruker EDS System. The AFM images were obtained using a Bruker Multi Mode 8 scanning probe microscope (SPM, VEECO) in tapping mode. The XPS analysis was performed using an ESCALAB 250 spectrometer (Thermo Fisher Scientific) with monochromated Al-Kα radiation (1486.6 eV) under a pressure of 2×10^{-9} Tor. FTIR was conducted by Bruker VERTEX 33 units in the wavenumber range of 400 - 4000 cm^{-1}. For the mechanical testing, the membranes were cut into strips (50 mm×5 mm). The tensile tests were performed at a loading rate of 1 mm/min at room temperature by using an Instron-5565 universal testing machine (USA). The zeta potential of the membranes was measured using SurPASS 3 Electrokinetic Analyzer (Anton Paar, Germany) through streaming potential measurements. The electrolyte solution was 1 mM KCl. The membrane was first cut into rectangular samples (10 mm×20 mm) and fixed on the sample holders using double-sided adhesive tape. The samples were carefully rinsed with the measuring electrolyte before the measurement. The pH was automatically adjusted by the integrated titration unit using HCl and KOH, respectively. The BET data was derived from physical gas sorption and desorption measurements by using Micromeritics ASAP2460 device.

Electrical measurements

The free-standing KANF membrane was clamped between a custom-made two-compartment electrochemical cell to test the ion transport properties. Homemade Ag/AgCl electrodes were used for detecting the current-voltage (*I-V*) responses the testing membrane. It should be noted that the testing membrane area was about 0.03 mm^2, the same as previous reports.[2-7] The *I-V* curves were recorded by using a Keithley 2450 source meter (Keithley Instruments). The range of the sweeping voltage was -1 V to +1 V, with a step voltage of 0.2V. The testing solutions were all prepared using ultrapure water (18.2 MΩ cm). For the long-term stability test, the membrane was clamped in the electro-chemical cell and stayed in the testing solutions all the time, and the testing solutions were replenished before each measurement.

Ion selectivity of KANF

The transference number t_n is calculated following the equation S1.[6, 8]

$$t_+ = \frac{1}{2}\left(\frac{E_{\mathrm{diff}}}{\frac{RT}{zF}\ln\frac{\lambda_{C_H} C_H}{\lambda_{C_L} C_L}} + 1 \right) \qquad (1)$$

where t_+ is the cation transference number; E_{diff} refers to the diffusion potential; *R, T, Z, F*, refer to the gas constant, temperature, valence charge and Faraday constant respectively; γ and c refer to ion activity coefficient and concentration.

Electrochemical energy conversion efficiency

Energy conversion efficiency energy conversion efficiency is defined as the ratio of the output energy (electrical energy) to the input energy (Gibbs free energy of mixing), the efficiency corresponding to the maximum power generation, $\eta_{\mathrm{max}\ W}$, is defined as equation S2.[3]

$$\eta_{\mathrm{max}} = \frac{(2t_n - 1)^2}{2} \qquad (2)$$

Statistical Analysis

Each experiment was repeated three times. All graphical data are reported as mean ±SD. The statistical analysis was performed by using the SPSS software (version 26.0, IBM SPSS, IL, USA). All results were analyzed by the two-tailed *t*-test, where $p<0.05$ was considered statistically significant.

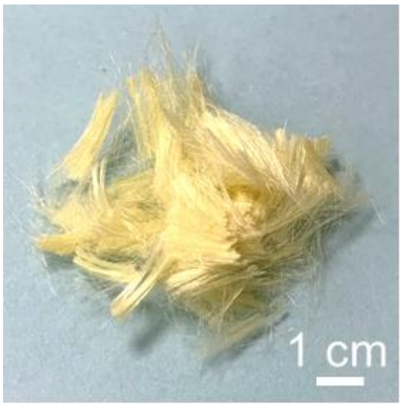

Figure S1. Photograph of the Kevlar yarns.

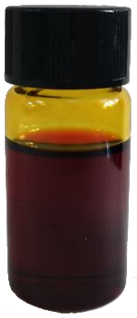

Figure S2. Photograph of the KANF solution.

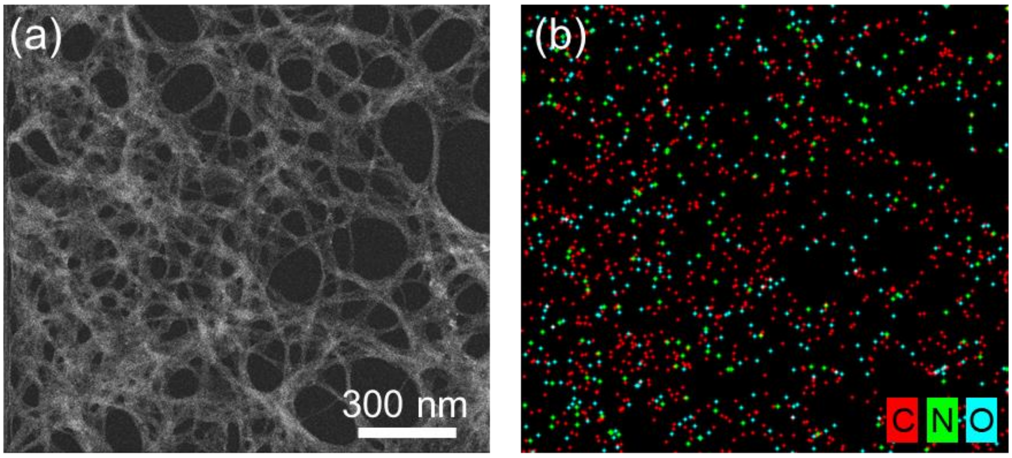

Figure S3. (a) TEM image and (b) the corresponding elemental mapping of the KANF.

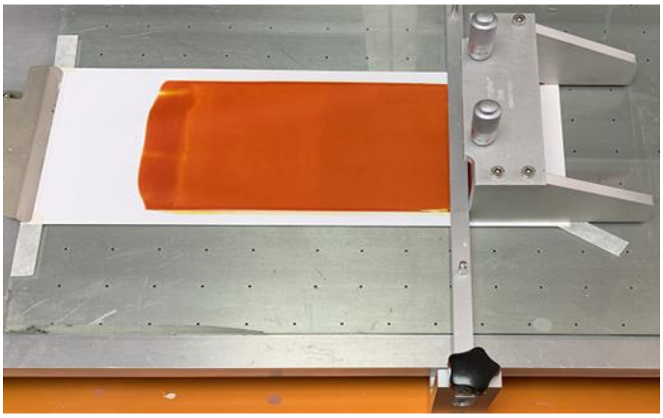

Figure S4. The device used to cast the KANF membrane.

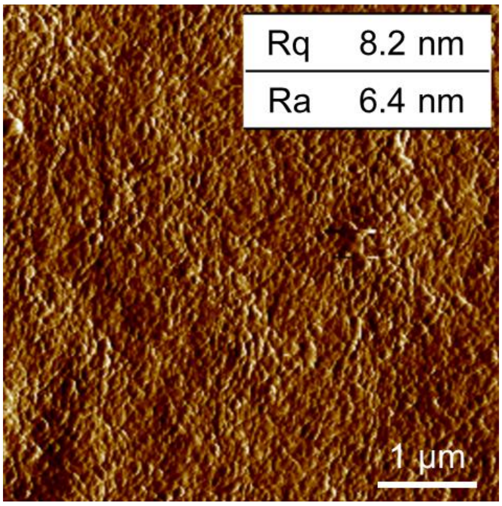

Figure S5. 3D AFM image and the surface roughness of the KANF membrane surface with a scan area of 5 μm × 5 μm. Inset shows the surface roughness. Rq, the maximum height of the profile. Ra, arithmetical mean deviation of the profile.

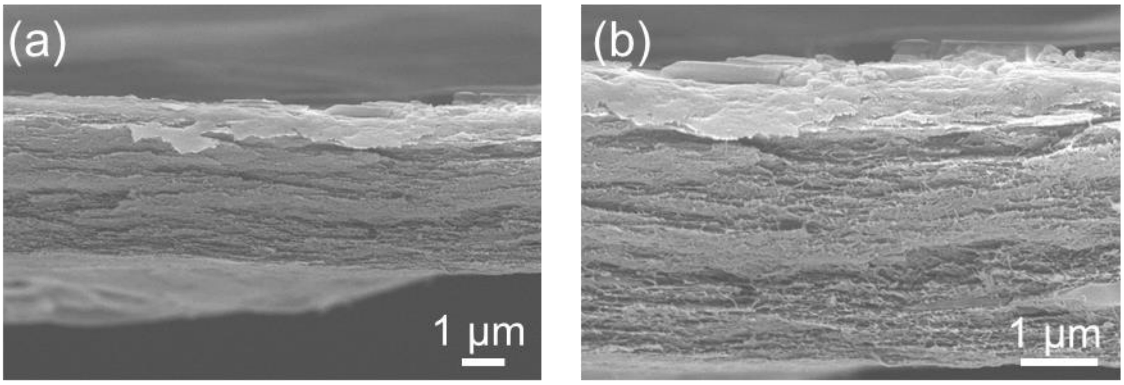

Figure S6. Cross-sectional SEM images of the KANF membrane with inter-connected channels.

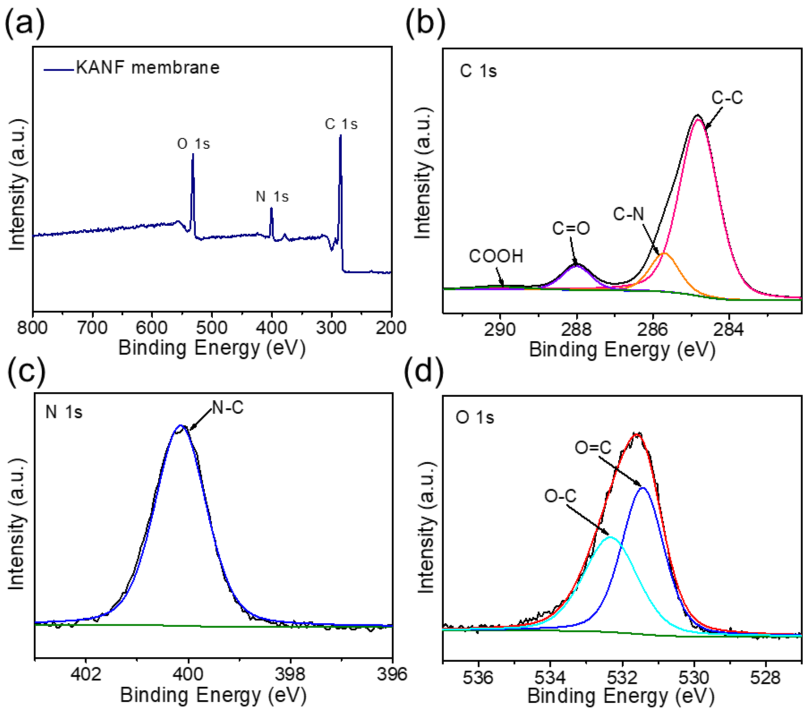

Figure S7. The XPS analysis presents the chemical states of the elements on the surface of the KANF membrane. (a) The survey spectrum, (b-d) the high-resolution spectra for C, N and O, respectively.

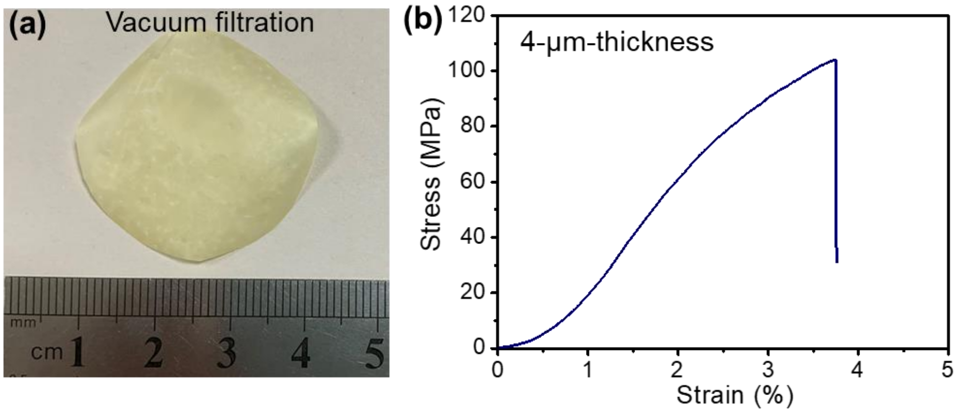

Figure S8. (a) Photograph of the vacuum filtrated KANF membrane. (b) Tensile stress-strain curve filtrated KANF membrane with a thickness of ~4 μm.

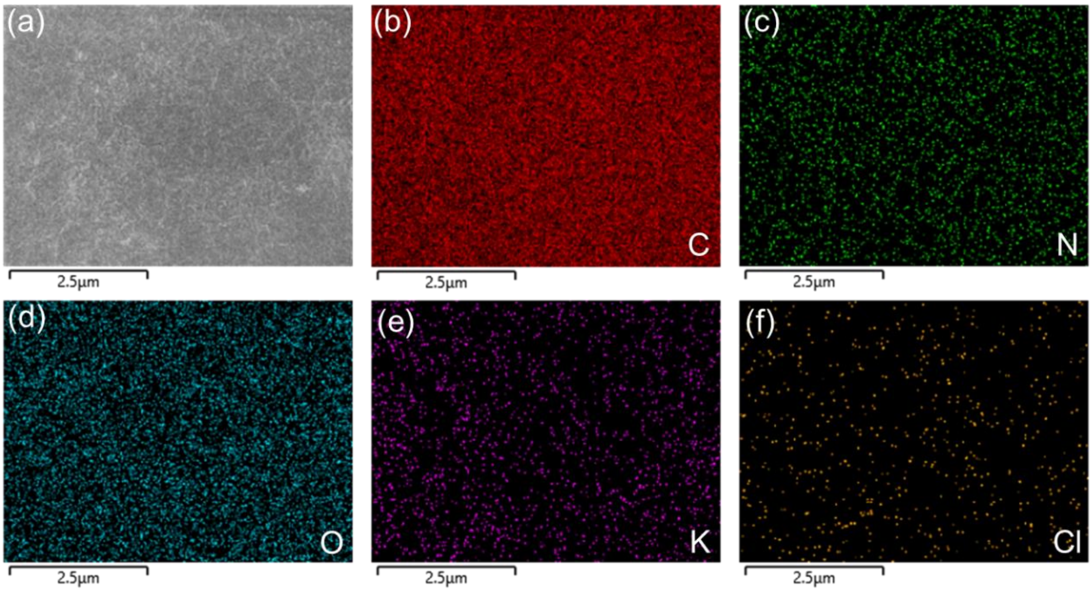

Figure S9. SEM image and corresponding elemental mappings of the KANF membrane after soaking in KCl solution (0.1 M). K shows the stronger signal than Cl demonstrated the cation-selective of the KANF membrane.

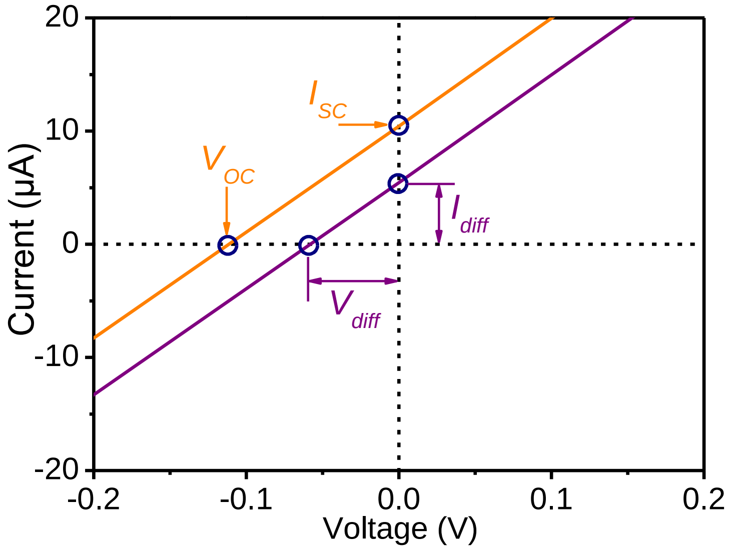

Figure S10. Current-voltage curve of KANF membrane under a 50-fold concentration gradient (c_{high} = 0.5 M, c_{low} = 0.01 M). The contribution from the redox potential has been subtracted, generating the purple line that represents the diffusion potential.[2]

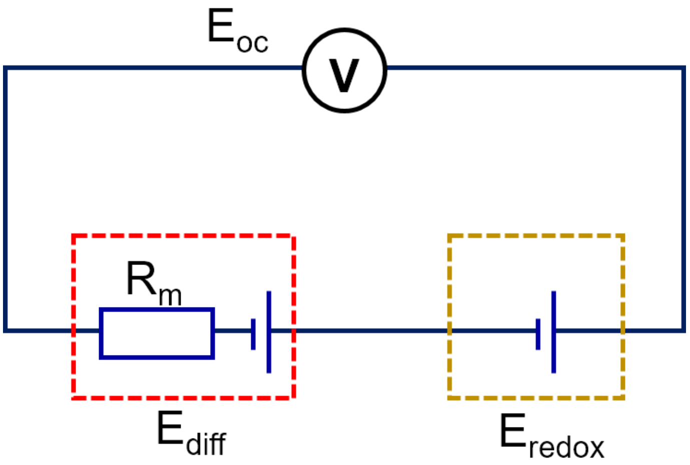

Figure S11. The equivalent circuit of the energy conversion system. The measured open-circuit voltage (E_{oc}) is composed of the diffusion potential (E_{diff}) contributed by the membranes and the redox potential (E_{redox}) on the electrode.

Scanning *I*-*V* curves were conducted under a series of concentration gradients to investigate the energy conversion performance of the membranes. According to the previous study,[2, 9] as shown in the basic equivalent circuit in Figure S11, the measured E_{oc} contains redox potential (E_{redox}) and diffusion potential (E_{diff}). The redox potential is derived from the unequal potential drop at the interface of electrode-electrolyte, while the diffusion potential is generated by the membrane-based power source. The diffusion potential (E_{diff}) can be calculated as $E_{diff} = E_{oc} - E_{redox}$. In order to measure the redox potential on the electrodes, a nonselective silicon pore with the same area was used instead of KANF membrane, in which only the redox reactions on the electrodes contribute to the measured voltage. The detailed information of E_{oc}, E_{redox}, and E_{diff} are listed in Table S1.

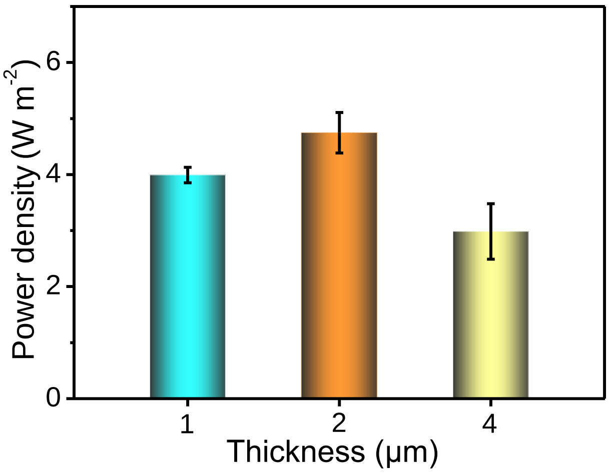

Figure S12. The output power densities of the KANF membrane-based generator with the thickness of 1 μm, 2 μm, and 4 μm that measured in synthetic seawater (0.5 M NaCl) and river water (0.01 M NaCl), respectively. Error bars represent s.d.

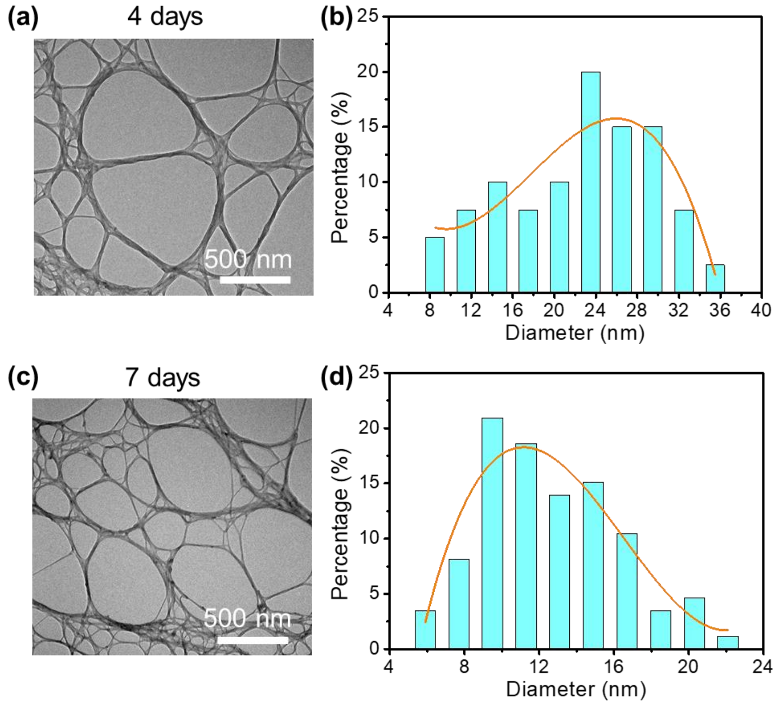

Figure S13. TEM images of the KANFs with different time of treatment (4 days and 7 days) and the corresponding diameter distributions.

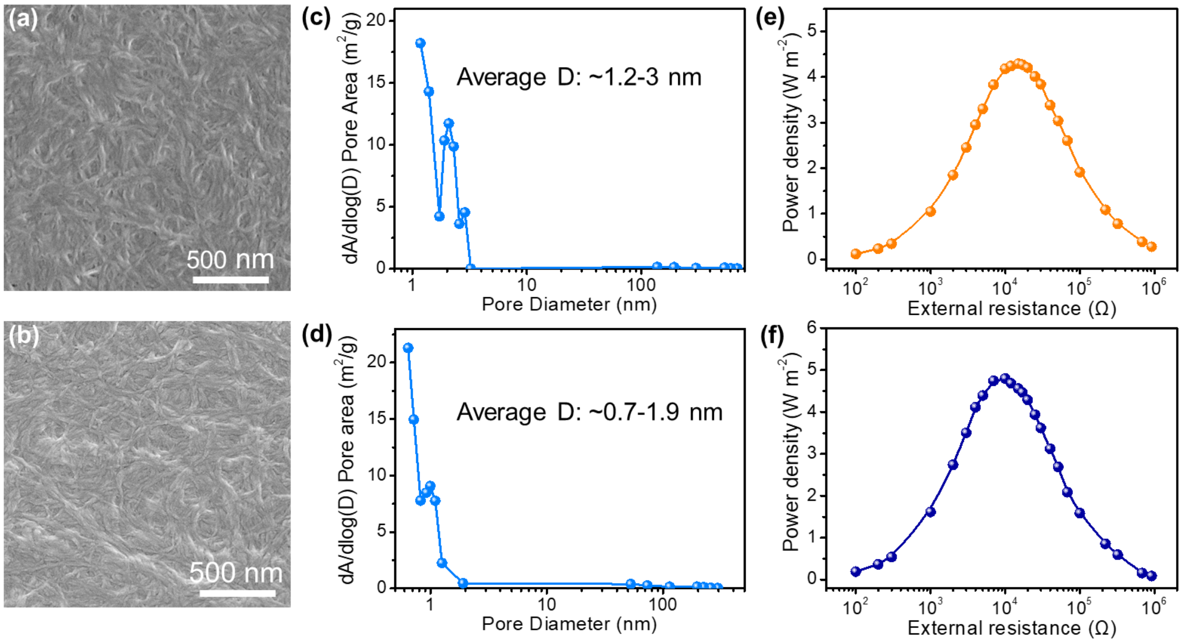

Figure S14. (a) and (b) Surface SEM images of KANF membrane with the the treating time of 4 days (M4) and 7 days (M7), respectively. (b) and (d) The pore diameter distribution of M4 and M7, respectively. (c) Output power density of M4 and M7-based generator, respectively.

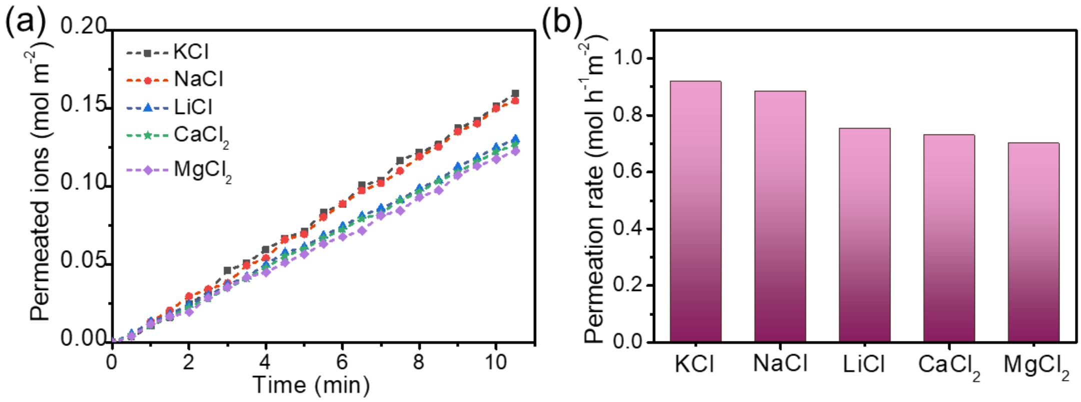

Figure S15. The ion permeation characteristics through KANF membranes. (a) The number of ions permeated through the KANF membranes (2-μm-thickness) against time for 0.2 M feed solutions. (b) The permeation rates of different cations through the KANF membranes.

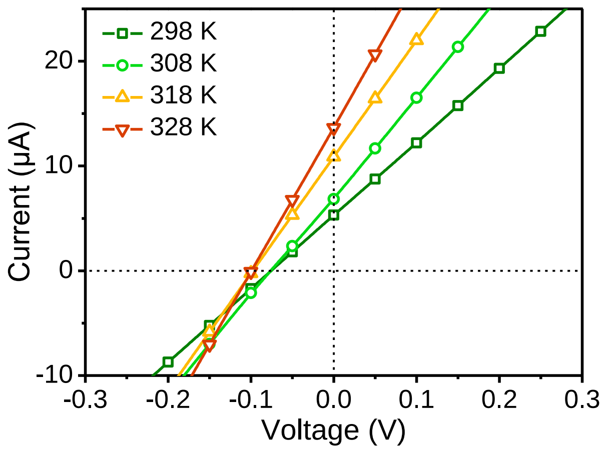

Figure S16. Current-voltage curve of KANF membrane under a 50-fold concentration gradient (C_{high} = 0.5 M, C_{low} = 0.01 M) under different temperatures. The redox potential of electrodes at 298K, 308 K, 318 K and 328K are 54 mV, 55 mV, 57 mV and 58 mV, respectively, which are less affected by temperature.

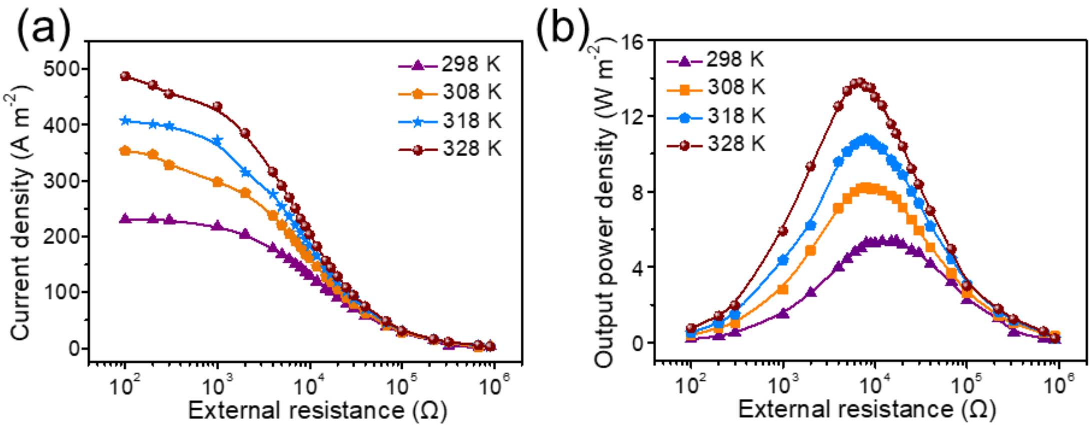

Figure S17. (a) Current density and (b) output power density of the KANF membrane as a function of the increasing external resistance under different temperatures.

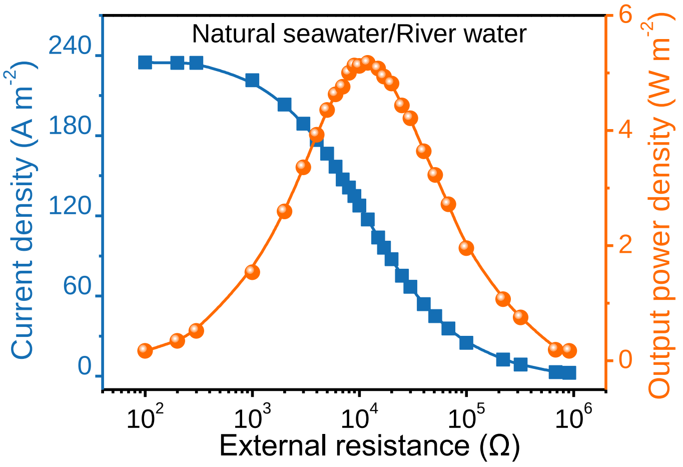

Figure S18. Current densities and power density of KANF membrane-based generator under nature seawater (*South China Sea*; ~0.58 M NaCl) and river water (*Pearl River*; ~0.006 M NaCl).

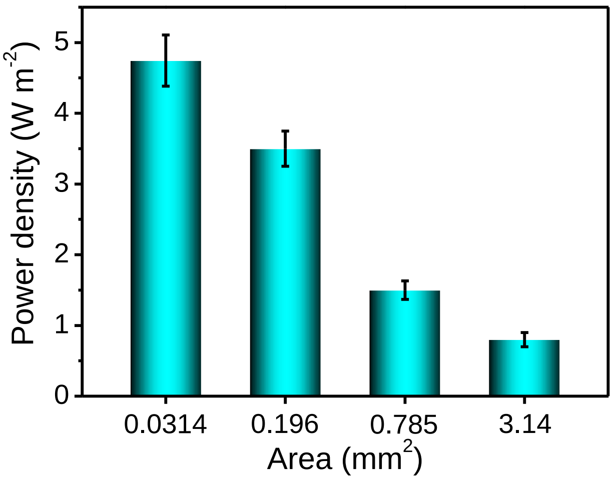

Figure S19. Comparison of testing area of KANF membranes for measured power density. The salinity gradient is under 0.5 M/0.01 M NaCl.

Table S1. The calculated E_{oc}, E_{redox} and E_{diff} under a series concentration gradient. Except for the combination of 0.5/0.01 M NaCl, the low concentration side of the others is fixed as 10^{-4} M KCl.

V (mV)	10-fold	30-fold	100-fold	300-fold	1000-fold	0.5/0.01
E_{oc}	85	115	157	196	224	121
E_{redox}	28	34	48	62	73	54
E_{diff}	57	81	109	134	151	67

Table S2. The calculated E_{diff}, t_+ and η under different concentration gradients. η is calculated from equation S2.

C_{high} (M)	C_{low} (M)	E_{diff} (mV)	t_+	η (%)
10^{-3}	10^{-4}	57	0.982	46.4
3×10^{-3}	10^{-4}	81	0.963	43.0
10^{-2}	10^{-4}	109	0.961	42.4
3×10^{-2}	10^{-4}	134	0.957	41.8
10^{-1}	10^{-4}	151	0.925	36.2
0.5	0.01	67	0.833	22.2

Table S3. Energy conversion performances of the KANF membranes compared with state-of-the-art membranes.

	Membrane	HC/LC	Thickness (μm)	P_{max} (W m^{-2})	Ref.
1	**silk-based hybrid membranes**	0.5 M/0.01 M NaCl	65	2.86	[3]
2	**Janus 3D porous membrane**	0.5 M/0.01 M NaCl	11	2. 66	[6]
3	**polymer/MOF**	0.5 M/0.01 M NaCl	85	2.87	[10]
4	**Oppositely charged graphene oxide membrane**	0.5 M/0.01 M NaCl	10	0.77	[11]
5	**Polymeric-C_3N_4 membrane**	0.1 M/0.1 mM KCl	0.25	0.21	[12]
6	**GO/CNFs membrane**	0.5 M/0.01 M NaCl	9	4.19	[13]
7	**GO/SNF/GO**	0.5 M/0.01 M NaCl	10	5.07	[14]
8	**oxidation of black phosphorus**	0.5 M/0.01 M NaCl	10	1.6	[4]
9	**graphene oxide/black phosphorus**	0.5 M/0.01 M NaCl	10	4.7	[4]
10	**2D kaolinite**	100	25	0.18	[15]
11	**BCP/PETM**	0.5 M/0.01 M NaCl	13.5	0.35	[16]
12	**BCP/AAOM**	500	60.1	1.67	[17]
13	**MS/AAO**	0.5 M/0.01 M NaCl	60.1	4.5	[18]
14	**Three-dimensional (3D) hydrogel**	0.5 M/0.01 M NaCl	210	3.9	[5]
15	**Heterogeneous lonomer/AAO**	0.5 M/0.01 M NaCl	99.2	3.15	[19]
16	**Mesoporous carbon/alumina**	0.5 M/0.01 M NaCl	64.2	3.46	[20]
17	**Vertical channel of MXene membrane**	0.5 M/0.01 M NaCl	10	4.6	[21]
18	**MXene/Kevlar nanofiber composite**	0.5 M/0.01 M NaCl	4.5	3.7	[2]
19	**MXene/boron nitride**	0.5 M/0.01 M NaCl	10	2.3	[22]
20	**Free-standing KANF membrane**	0.5 M/0.01 M NaCl	1	4.0	**This work**
	**Free-standing KANF membrane**	0.5 M/0.01 M NaCl	2	4.8	**This work**
	**Free-standing KANF membrane**	0.5 M/0.01 M NaCl	4	2.9	**This work**

Table S4. Main cost of raw materials for KANF membrane fabrication.

Raw material	Needed amount for 1 m^2 membrane	Unit price	Cost of raw material for 1 m^2 membrane (RMB)
KOH	2 g	36 RMB/kg	0.072
DMSO	100 ml	10 RMB/L	1
Kevlar	2 g	200 RMB/kg	0.4
**Total cost of raw materials for 1 m^2 membrane**			1.472

References

[1] X. Li, H.-C. Li, T.-T. You, Y.-Y. Wu, S. Ramaswamy, F. Xu, *Ind. Crop. Prod.* **2019**, *140*, 111603.

[2] Z. Zhang, S. Yang, P. Zhang, J. Zhang, G. Chen, X. Feng, *Nat. Commun.* **2019**, *10*, 2920.

[3] W. Xin, Z. Zhang, X. Huang, Y. Hu, T. Zhou, C. Zhu, X.-Y. Kong, L. Jiang, L. Wen, *Nat. Commun.* **2019**, *10*, 3876.

[4] Z. Zhang, P. Zhang, S. Yang, T. Zhang, M. Loeffler, H. Shi, M. R. Lohe, X. Feng, *P. Natl. Acad. Sci.* **2020**, *117*, 13959.

[5] Z. Zhang, L. He, C. Zhu, Y. Qian, L. Wen, L. Jiang, *Nat. Commun.* **2020**, *11*, 875.

[6] X. Zhu, J. Hao, B. Bao, Y. Zhou, H. Zhang, J. Pang, Z. Jiang, L. Jiang, *Sci. Adv.* **2018**, *4*, eaau1665.

[7] C. Li, L. Wen, X. Sui, Y. Cheng, L. Gao, L. Jiang, *Sci. Adv.* **2021**, *7*, eabg2183.

[8] X. Liu, M. He, D. Calvani, H. Qi, K. B. S. S. Gupta, H. J. M. de Groot, G. J. A. Sevink, F. Buda, U. Kaiser, G. F. Schneider, *Nat. Nanotechnol.* **2020**, *15*, 307.

[9] Y. Zhou, L. Jiang, *Joule* **2020**, *4*, 2244.

[10] R. Li, J. Jiang, Q. Liu, Z. Xie, J. Zhai, *Nano Energy* **2018**, *53*, 643.

[11] J. Ji, Q. Kang, Y. Zhou, Y. Feng, X. Chen, J. Yuan, W. Guo, Y. Wei, L. Jiang, *Adv. Funct. Mater.* **2017**, *27*, 1603623.

[12] K. Xiao, P. Giusto, L. Wen, L. Jiang, M. Antonietti, *Angew. Chem. Int. Ed.* **2018**, *57*, 10123.

[13] Y. Wu, W. Xin, X.-Y. Kong, J. Chen, Y. Qian, Y. Sun, X. Zhao, W. Chen, L. Jiang, L. Wen, *Mater. Horizons* **2020**, *7*, 2702.

[14] W. Xin, H. Xiao, X.-Y. Kong, J. Chen, L. Yang, B. Niu, Y. Qian, Y. Teng, L. Jiang, L. Wen, *ACS Nano* **2020**, *14*, 9701.

[15] H. Cheng, Y. Zhou, Y. Feng, W. Geng, Q. Liu, W. Guo, L. Jiang, *Adv. Mater.* **2017**, *29*, 1700177.

[16] Z. Zhang, X.-Y. Kong, K. Xiao, Q. Liu, G. Xie, P. Li, J. Ma, Y. Tian, L. Wen, L. Jiang, *J. Am. Chem. Soc.* **2015**, *137*, 14765.

[17] X. Sui, Z. Zhang, C. Li, L. Gao, Y. Zhao, L. Yang, L. Wen, L. Jiang, *ACS. Appl. Mater. Inter.* **2019**, *11*, 23815.

[18] S. Zhou, L. Xie, L. Zhang, L. Wen, J. Tang, J. Zeng, T. Liu, D. Peng, M. Yan, B. Qiu, Q. Liang, K. Liang, L. Jiang, B. Kong, *ACS. Appl. Mater. Inter.* **2021**, *13*, 8782.

[19] T. Xiao, Q. Zhang, J. Jiang, J. Ma, Q. Liu, B. Lu, Z. Liu, J. Zhai, *Energy Technol.* **2019**, *7*, 1800952.

[20] J. Gao, W. Guo, D. Feng, H. Wang, D. Zhao, L. Jiang, *J. Am. Chem. Soc.* **2014**, *136*, 12265.

[21] L. Ding, D. Xiao, Z. Lu, J. Deng, Y. Wei, J. Caro, H. Wang, *Angew. Chem. Int. Ed.* **2020**, *59*, 8720.

[22] G. Yang, D. Liu, C. Chen, Y. Qian, Y. Su, S. Qin, L. Zhang, X. Wang, L. Sun, W. Lei, *ACS Nano* **2021**, *15*, 6594.
